# Age-Related Alterations Affecting the Chondrogenic Differentiation of Synovial Fluid Mesenchymal Stromal Cells in an Equine Model

**DOI:** 10.3390/cells8101116

**Published:** 2019-09-20

**Authors:** Eleonora Mazzotti, Gabriella Teti, Mirella Falconi, Francesca Chiarini, Barbara Barboni, Antonio Mazzotti, Aurelio Muttini

**Affiliations:** 1 Faculty of Bioscience and Agro-Food and Environmental Technology, University of Teramo, 64100 Teramo, Italy; 2 Department of Biomedical and Neuromotor Sciences, University di Bologna, 40126 Bologna, Italy; 3 CNR-National Research Council of Italy, Institute of Molecular Genetics “Luigi Luca Cavalli-Sforza”, Unit of Bologna, 40136 Bologna, Italy; 4 IRCCS Istituto Ortopedico Rizzoli, 40136 Bologna, Italy; 5 1st Orthopedic and Traumatologic Clinic, IRCCS Istituto Ortopedico Rizzoli, Via Giulio Cesare Pupilli 1, 40136 Bologna, Italy; 6 Stem TeCh Group, 66100 Chieti, Italy

**Keywords:** aging, senescence, MSCs, chondrogenic differentiation, osteoarthritis

## Abstract

Osteoarthritis is a degenerative disease that strongly correlates with age and promotes the breakdown of joint cartilage and subchondral bone. There has been a surge of interest in developing cell-based therapies, focused particularly on the use of mesenchymal stromal cells (MSCs) isolated from adult tissues. It seems that MSCs derived from synovial joint tissues exhibit superior chondrogenic ability, but their unclear distribution and low frequency actually limit their clinical application. To date, the influence of aging on synovial joint derived MSCs’ biological characteristics and differentiation abilities remains unknown, and a full understanding of the mechanisms involved in cellular aging is lacking. The aim of this study was therefore to investigate the presence of age-related alterations in synovial fluid MSCs and their influence on the potential ability of MSCs to differentiate toward chondrogenic phenotypes. Synovial fluid MSCs, isolated from healthy equine donors from 3 to 40 years old, were cultured in vitro and stimulated towards chondrogenic differentiation for up to 21 days. An equine model was chosen due to the high degree of similarity of the anatomy of the knee joint to the human knee joint and as spontaneous disorders develop that are clinically relevant to similar human disorders. The results showed a reduction in cell proliferation correlated with age and the presence of age-related tetraploid cells. Ultrastructural analysis demonstrated the presence of morphological features correlated with aging such as endoplasmic reticulum stress, autophagy, and mitophagy. Alcian blue assay and real-time PCR data showed a reduction of efficiency in the chondrogenic differentiation of aged synovial fluid MSCs compared to young MSCs. All these data highlighted the influence of aging on MSCs’ characteristics and ability to differentiate towards chondrogenic differentiation and emphasize the importance of considering age-related alterations of MSCs in clinical applications.

## 1. Introduction

Many joint disorders, such as osteoarthritis, can lead to cartilage defects. Articular cartilage shows limited regenerative potential because of its avascular nature and due to its paucity of mesenchymal stromal cells (MSCs). For this reason, articular cartilage defects remain a real challenge in the field of regenerative medicine [1]. A great deal of effort has been made regarding cell therapy based on the use of MSCs, often in combination with biomaterials, in order to improve tissue regeneration. Currently, MSCs still represent a good candidate for tissue repair and regeneration [2,3]. MSCs or mesenchymal progenitor cells can be defined as non-hematopoietic, self-renewing cells that can be expanded ex vivo and induced to terminally differentiate, under certain conditions, into cells of mesenchymal lineage, including osteoblasts [4,5], chondrocytes [6,7,8], adipocytes [9], and hematopoietic-supporting stroma [10]. 

Adult MSCs are localized in a special microenvironment, called the “niche”, that provides homeostasis, proliferative control, and the maintenance of cell number [11]. Since their first isolation from bone marrow [12,13], MSCs have been described in several other tissues, both from adult and fetal tissues, such as adipose tissue [14], dental pulp [15], periosteum [16,17], the umbilical cord [18,19], human synovial membrane (SmMSCs) [20], as well as in synovial fluid (synovial fluid mesenchymal stromal cells (SfMSCs)) [1]. 

MSCs from different tissues have been shown to differ dramatically in their chondrogenic potency. Actually, it is considered that SfMSCs exhibit a superior chondrogenic ability compared with those derived from non-joint tissues [1]. The immunophenotype of SfMSC recapitulates a typical mesenchymal profile and displays a positive expression of CD105, CD90, and CD44, and the cells are negative for CD45 [21]. They are capable of differentiating into chondrocytes, osteoblasts, and adipocytes [22]. The numbers of SfMSCs from anterior cruciate ligament injury patients were 100 times higher than those from healthy knees [23], and their number also increased in knees with degenerated cartilage and osteoarthritis [22,23,24]. Although a growing body of research suggests that SfMSCs and SmMSCs represent a promising stem cell source for osteochondral repair, only a few experimental studies have demonstrated that SmMSCs can be effective in resurfacing full-thickness [25] or partial-thickness cartilage defects in rabbits [26]. Additionally, only a few papers have been published to date demonstrating the presence and the multipotence of SfMSCs isolated from the synovial fluid of normal and pathological horse joints [27]. 

Despite several powerful applications of MSCs in regenerative medicine, their features and proprieties can be strongly influenced by several factors, such as aging. Aging is a complex process that affects most of the biological functions, generally ending in diseases and death, due to the accumulation of different types of stress [28]. MSCs isolated from young and elderly individuals have been shown to have different properties, such as a reduction of colony forming efficiency, proliferative ability, and osteogenic potential [29]. Indeed, multiple influences of age on stem cell function have been confirmed, including effects on stem cell number, proliferation potential, senescence and apoptosis, replicative capacity, and in vitro differentiation potential [30]. MSCs isolated from adipose tissue have exhibited a donor’s age influence on replicative senescence, leading to cell apoptosis [31]. Furthermore, it was demonstrated that chondrogenic and osteogenic plasticity were strongly age-related, in a different manner to adipogenic and neurogenic plasticity [31]. On the contrary, dermal fibroblasts display a steady proliferative potential that is totally independent of donor age [32]. 

Considering the presence of contradictory information regarding the influence of aging on MSCs’ biological performance and the importance of examining their proprieties to develop relevant stem cell therapeutic strategies for regenerative medicine, the aim of this study is to investigate the age-related influence on synovial fluid MSC phenotypes and on their in vitro chondrogenic differentiation potential. 

SfMSCs were isolated from healthy tibio–tarsal joints of horses ranging from 3 to 40 years old, and they were cultured in vitro and characterized for cell proliferation assay, cell cycle analysis, actin F protein staining, and ultrastructure analysis with the aim of highlighting the presence of age-related alterations. To evaluate the influence of aging on chondrogenic differentiation, SfMSCs were stimulated in vitro for up to 21 days with chondrogenic factors, the expression of chondrogenic markers was determined, and an Alcian blue staining assay was conducted.

An equine model was chosen due to the high similarity of the anatomy and the biomechanics to the human knee joint. Moreover, horses suffer from cartilage injuries and joint diseases such as osteoarthritis and osteochondrosis that are clinically relevant to similar human disorders [33].

## 2. Materials and Methods

### 2.1. Isolation of Equine SfMSCs

Sample collection procedures were approved by the ethical committee of the University of Teramo (3/10/2018) and by the Italian Ministry of Health (approval No. 0018640-P-2/0772018). After obtaining the informed consent from the owners, synovial fluid (Sf) was aseptically collected from healthy tibio–tarsal joints of horses ranging from 3 to 40 years old. The age, breed, sex, and the number of samples are summarized in Table 1. Samples were collected in four groups, depending on age: 3 years old (3Y), 12 years old (12Y), 23 years old (23Y), and 40 years old (40Y). The presence of any joint disease was previously ruled out by means of clinical and radiographical examination. A minimum of 5 mL of clear and blood-free Sf was obtained from each tibio–tarsal joint. Sf was diluted with phosphate buffered saline (PBS) up to five volumes and was subsequently filtered through a 70 μm nylon filter (Cell Strainer, BD Falcon, Leipzig, Germany) to remove debris and centrifuged at 160 *g* for 5 min at room temperature (RT). 

The pellets were resuspended in Dulbecco’s modified Eagle’s medium (DMEM, Life Technologies, Monza, Italy), supplemented with 10% fetal bovine serum (FBS, Life Technologies, Monza, Italy), and plated in a 25 cm^2^ culture flask, at 37 °C in 5% CO_2_ for 7 days. Cells adhering to the bottom of the flask were subsequently detached and cultured in the same conditions. Briefly, cells from passage 1 to passage 6 were utilized for the all the experiments of the study.

### 2.2. mRNA Expression of Mesenchymal and Self-Renewal Markers by RT-PCR and Real-Time PCR

Total RNA was obtained with an RNeasy Mini Kit (Invitrogen, Thermo Fisher Scientific, Waltham, MA, USA) starting from cellular pellets, and 100 ng of total RNA was reverse transcribed into first-strand cDNA using SuperScript™ III One-Step RT-PCR System (Invitrogen, Thermo Fisher Scientific, Waltham, MA, USA). The PCR was performed by Phusion Green Hot Start II High-Fidelity DNA Polymerase kit (Thermo Fisher Scientific, Waltham, MA, USA). Equine glyceraldehyde-3-phosphate dehydrogenase gene (*GAPDH*) was used as a housekeeping gene for amplification control during the PCR assay. The primer sequences were designed by Primer design Software (Primer3-based OligoPerfect, Thermo Fisher Scientific, Waltham, MA, USA). The amplified DNA was then electrophoresed on a 2% agarose gel and visualized by ethidium bromide staining; images were acquired by Image Station 2000R (Kodak, NY, USA). The primer sequences used are shown in Table 2. 

For mRNA quantification, a Powerup SYBR master mix kit (Life Technologies, Thermo Fischer Scientific, Monza, Italy) was used in combination with the specific primers shown in Table 2. Relative gene expression levels were normalized to those of equine glyceraldehyde 3-phosphate dehydrogenase. Data are presented as fold changes relative to levels of the reference sample by using formula 2^−ΔΔCT^, as recommended by the manufacturer (User Bulletin number 2 P/N 4303859; Applied Biosystems, Waltham, MA, USA) The results were expressed as fold changes compared to a reference control group made of a pool four healthy equine subjects, each 3 years old.

### 2.3. BrdU Proliferation Assay

Cell proliferation was assayed by BrdU assay kit (Roche, Basel, Switzerland) according to the manufacturer’s instructions. Briefly, cells were seeded into a 96-well culture plate with DMEM containing 10% FBS, 1% penicillin and streptomycin, and 1% fungizone in a density of 10^4^ cells/well. After 24 h, the medium was changed to a fresh one containing 10 μM BrdU for 12 h at 37 °C. After the removal of the labeling solution, cells were fixed and incubated with anti-BrdU conjugated with peroxidase antibody for 90 min at RT. After three washing steps in PBS, a tetramethyl-benzidine (TMB) substrate solution was added for 10 min at RT, and the reaction was stopped with 1 M H_2_SO_4_. The optical density was measured using a spectrophotometer microplate reader (Model680, BioradLab Inc., CA, USA) at a wavelength of 450 nm and a reference wavelength of 690 nm. 

Data from SfMSCs isolated from the groups of 12Y, 23Y, and 40Y were expressed as the percentage of relative proliferation compared to SfMSCs isolated from a pool of four different healthy equine subjects, each 3 years old (reference control group).

### 2.4. Cell Cycle Analysis

The flow cytometric analysis of the cell cycle was performed using propidium iodide (PI)/RNase A staining according to standard procedures, as described previously [34]. Briefly, the SfMSCs were detached from the surface of the flask with enzyme digestion for 3 min at room temperature, and then collected and centrifuged at 300 *g* for 5 min. The pellets were resuspended in PBS and counted by a hemocytometer. A total of 2.5 × 10^5^ cells was centrifuged and then resuspended in ethanol 70% overnight at −20 °C. Cells were then centrifuged at 300 *g* for 5 min, and the pellets obtained were washed twice in PBS and resuspended in a solution of propidium iodide for at least 30 min. Samples were analyzed on a FC500 flow cytometer (Beckman Coulter, Indianapolis, IN, USA) with the appropriate software (version 2.2, CXP, Beckman Coulter). At least 15,000 events per sample were acquired.

### 2.5. Doubling Time Assay

SfMSCs isolated from all donors were seeded at the density of 5 × 10^3^ cells/cm^2^ in 25 cm^2^ flasks and allowed to grow until reaching 90% of confluence. At this point, cells were detached with enzyme digestion, counted by automated cell countess (Countess™ Automated Cell Counter, Invitrogen, Thermo Fisher Scientific, Waltham, MA, USA) and reseeded at passage P1 at the same density as previously described. The procedure was repeated from passage P1 to P4. Cell-doubling time (DT) was calculated from counts for each passage according to the following two formulae [34]:

CD = ln(Nf/Ni)/ln(2)

DT = CT/CD

where Nf and Ni are the final and initial number of cells, respectively, and CT is the cell incubation time expressed in days. 

### 2.6. F-Actin Staining

F-actin protein was labeled by fluorescent phallotoxin, a bicyclic peptide showing a high binding affinity toward actin small filament. The staining procedure was performed according to the manufacturer’s instructions (Molecular Probes, Invitrogen, Eugene, OR, USA). Briefly, for each sample, 2 × 10^5^ cells were seeded on cover glasses in MEM supplemented with 10% FBS at 37 °C for 24 h. Then, samples were fixed in 4% paraformaldehyde in PBS for 20 min at 4 °C and subsequently permeabilized by 0.1% Triton-X in PBS for 5 min at RT. Coverslips were stained with fluorescent phallotoxin diluted to 1:40 in 1% bovine serum albumin (BSA) for 20 min at RT. After three washes in PBS and distilled water, coverslips were counterstained with 4′,6-diamidino-2-phenylindole (DAPI) and then mounted with the permanent mountant ProLong gold (Invitrogen, Thermo Fisher Scientific, Waltham, MA, USA). Images were acquired by the fluorescence microscope Eclipse E800 (Nikon, Tokyo, Japan). 

The quantitative analysis of phallotoxin-stained areas was assessed by area, counting five fields for each of the three slides per sample at 60× magnification by the Leica Qwin 3.0 software (Leica Microsystems Srl, Cambridge, UK), which allowed the phallotoxin-stained area to be selected and measured.

### 2.7. TEM Analysis

Monolayers of equine SfMSCs were fixed with 2.5% (v/v) glutaraldehyde in 0.1 M cacodylate buffer for 2 h at 4 °C and post-fixed with a solution of 1% osmium tetroxide in 0.1 M cacodylate buffer. The cells were then embedded in epoxy resins after a graded-acetone serial dehydration step. Ultrathin slices of 100 nm were stained by uranyl acetate solution and lead citrate and then observed with a transmission electron microscope, CM10 Philips (FEI Company, Eindhoven, The Netherlands), at an accelerating voltage of 80 kV. Images were recorded by Megaview III digital camera (FEI Company, Eindhoven, The Netherlands). 

### 2.8. Alcian Blue

SfMSCs isolated from all donors were seeded into a 6-well culture plate with DMEM containing 10% FBS, 1% penicillin and streptomycin, and 1% fungizone in a density of 10^5^ cells/well. After 24 h, the medium was changed with chondrogenic medium consisting of MEM supplemented with 2% FBS, 100 nM dexamethasone (Sigma-Aldrich, St. Louis, MO, USA), 100 μg/mL ascorbate-2-phosphate (Sigma-Aldrich, St. Louis, MO, USA), ITS (6.25 μg/mL insulin, 6.25 μg/ mL transferrin, 6.25 μg/mL selenous acid) (Gibco, Thermo Fisher Scientific, Monza, Italy), and 10 ng/mL of transforming growth factor β3 (TGF-β3) (Millipore, Milan, Italy) for 21 days at 37 °C and 5% CO_2._ Chondrogenic differentiation was induced up to 21 days, changing the medium every three days. At the end of the treatment, cells were immediately fixed in 4% formaldehyde in phosphate buffer (PBS) for 4 h at 4 °C and then processed for the Alcian blue staining procedure with an Alcian blue staining kit (Bio-Optica, Milan, Italy). Images were acquired by the light microscope Eclipse E800 (Nikon, Tokyo, Japan).

### 2.9. Chondrogenic Differentiation

SfMSCs were cultured in Dulbecco’s modified eagle medium (DMEM), supplemented with 10% fetal bovine serum (FBS) (Sigma-Aldrich, St. Louis, MO), and then they were grown into 3D cultures as micromasses. Briefly, 2.5 × 10^5^ cells were pelleted (10 min, 160 g) per tube (Sarstedt, Neumbrecht, Germany) and kept in 1 mL chondrogenic medium consisting of MEM supplemented with 2% FBS, 100 nM dexamethasone (Sigma-Aldrich, St. Louis, Missouri, USA), 100 μg/mL ascorbate-2-phosphate (Sigma-Aldrich, St. Louis, Missouri, USA), ITS (6.25 μg/mL insulin, 6.25 μg/ mL transferrin, 6.25 μg/mL selenous acid) (Gibco, Thermo Fisher Scientific, Monza, Italy), and 10 ng/mL of TGF-β3 (Millipore, Milan, Italy) for 21 days at 37 °C and 5% CO_2_. Control samples consisted of SfMSCs cultured in a micromass system in MEM supplemented with 2% FBS for up to 21 days. At the end of each treatment, cells were collected and RNA was extracted for quantitative real-time PCR (qRT-PCR) to evaluate the expression of *SOX9, COLL2,* and *ACAN* chondrogenic markers.

### 2.10. Real-Time PCR

The chondrogenic mRNA marker expression was analyzed via real-time PCR (7500 Applied Biosystems, Life Technologies, Monza, Italy). For mRNA quantification, the Powerup SYBR master mix kit (Life Technologies, Thermo Fischer Scientific, Monza Italy) was used in combination with the specific primers for the chondrogenic markers shown in Table 2. Relative gene expression levels were normalized to those of equine glyceraldehyde 3-phosphate dehydrogenase. Data are presented as fold changes relative to levels of the reference sample by using formula 2^−ΔΔCT^, as recommended by the manufacturer (User Bulletin number 2 P/N 4303859; Applied Biosystems). The quantitative analysis of mRNA expression was performed in triplicate for each donor. The results were expressed as fold changes compared to a reference control group made of a pool of four healthy equine subjects, each 3 years old.

### 2.11. Statistical Analysis

Statistical analysis was carried out using GRAPH PAD PRISM 5.0 software (San Diego, CA, USA) applying a one-way ANOVA followed by Tukey’s multiple comparison test. The differences were considered significant at *p* < 0.05.

## 3. Results

With the primary aim of demonstrating the presence of age-associated features in SfMSCs isolated from equine donors of different ages, experiments analyzing the mRNA expression of mesenchymal and self-renewal markers—cell proliferation, cell cycle, cell surface area, actin distribution, and cell ultrastructure—were carried out. Our data showed the presence of age-related features that could interfere with the biological proprieties of SfMSCs. Following the preliminary results, to demonstrate an age-related ability of SfMSCs to differentiate toward chondrogenic phenotypes, the expression of mRNA chondrogenic markers and the deposition of proteoglycans were analyzed after 21 days of in vitro stimulation with chondrogenic factors. 

### 3.1. Mesenchymal and Self-Renewal Expression Markers

In order to demonstrate the mesenchymal phenotype of SfMSCs, the expressions of *CD105*, *CD90*, and *CD34* markers were investigated in agreement with the minimal criteria of the International Society of Cellular Therapy (ISCT) [35]. Furthermore, the SfMSCs were further characterized by evaluating the expression of self-renewal markers, such as homeobox protein transcription factor (*NANOG*), octamer-binding transcription factor 4 (*OCT4*), and SRY (sex determining region Y)-box 2 (*SOX2*) genes [36].

**Figure 1:**
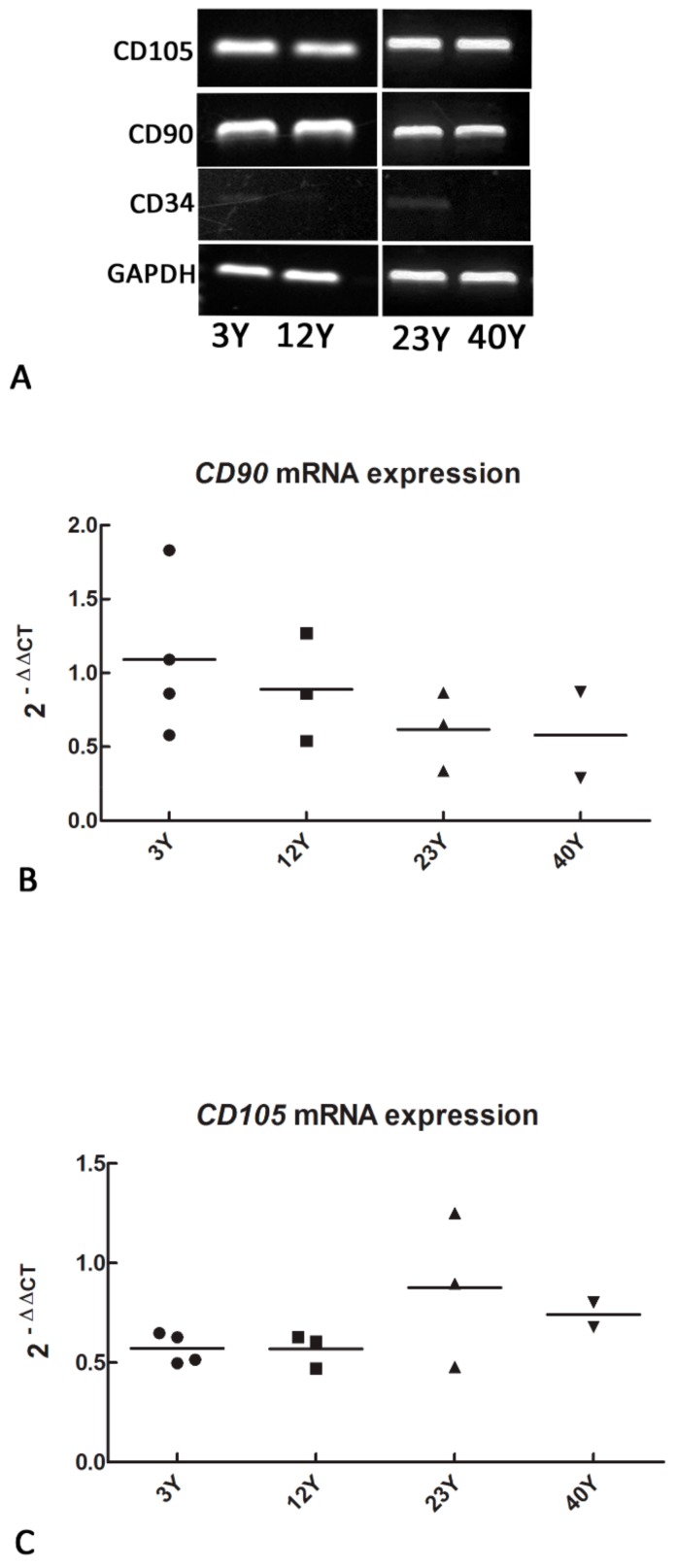
(**A**) RT-PCR analysis showing a positive expression of *CD105* and *CD90* and a negative expression of *CD34* mesenchymal stromal surface markers in synovial fluid mesenchymal stromal cells (SfMSCs) cultured from equine donors of different ages: 3Y (3 years old), 12Y (12 years old), 23Y (23 years old), and 40Y (40 years old). Representative images of three independent experiments; (**B**) real-time PCR data showing the quantitative expression of *CD90* mRNA. The value corresponding to each single donor and the mean level inside each group (black line) was reported. (**C**) Real-time PCR data showing the quantitative expression of *CD105* mRNA. The value corresponding to each single donor and the mean level inside each group (black line) was reported. The values are represented as fold increases related to a reference control consisting of a pool of four different healthy equine subjects, each 3 years old. No statistical differences were observed between groups.

A few days after primary culture, SfMSCs isolated from equine donors of 3Y, 12Y, 23Y, and 40Y groups adhered to plastic culture flasks. RT-PCR analysis showed a positive expression for *CD105* and *CD90* cell surface markers and a negative expression for the hematopoietic marker *CD34* (Figure 1A). Real-time PCR data showed that the relative quantifications of the mRNAs of *CD90* (Figure 1B) and *CD105* (Figure 1C) were not statistically different in all the samples analyzed, suggesting a lack of a relationship between a donor’s age and the quantitative expression of the mesenchymal stromal markers.

**Figure 2:**
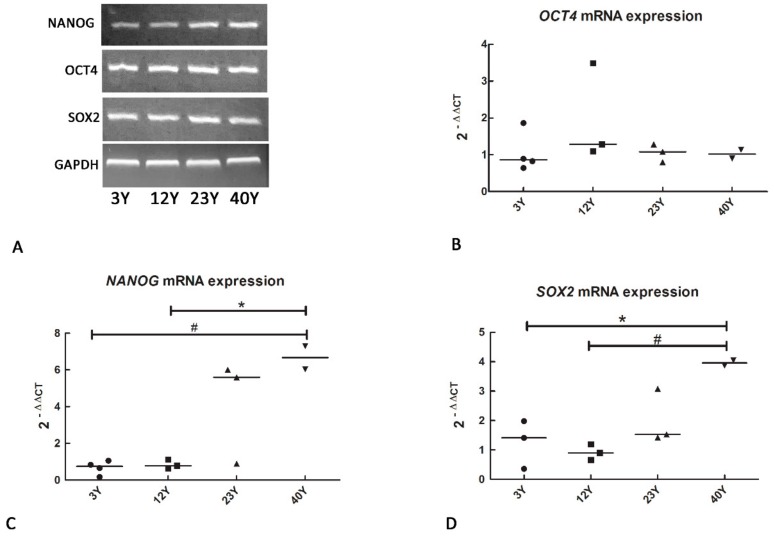
(**A**) RT-PCR analysis showing a positive expression of *NANOG*, *OCT4*, and *SOX2* self-renewal markers in SfMSCs cultured from equine donors of different ages: 3Y (3 years old), 12Y (12 years old), 23Y (23 years old), and 40Y (40 years old). Representative images of three independent experiments; (**B**) real-time PCR data showing the quantitative expression of *NANOG* mRNA in each single donor and the mean level inside each group (black line). No statistical differences were observed between groups. (**C**) Real-time PCR data showing the quantitative expression of *NANOG* mRNA in each single donor and the mean level inside each group (black line). * represents a significant difference between the 12Y and 40Y group; *p* < 0.05. # represents a significant difference between the 3Y and 40Y group; *p* < 0.05. (**D**) Real-time PCR data showing the quantitative expression of *SOX2* mRNA in each single donor and the mean level inside each group (black line). * represents a significant difference between the 3Y and 40Y group; *p* < 0.05. # represents a significant difference between the 12Y and 40Y group; *p* < 0.05.

Regarding self-renewal markers, all the isolated SfMSCs were positive for *NANOG*, *OCT4*, and *SOX2* mRNA expression (Figure 2A), suggesting no influence of aging on multipotency markers. More precisely, quantitative analysis by real-time PCR showed the lack of any significant difference regarding the mRNA expression of *OCT4* in all the samples tested (Figure 2B), while the mRNA expression of *NANOG* and *SOX2* was upregulated in the 40Y group compared to 3Y and 12Y (Figure 2C,D).

The values are represented as fold increases related to a reference control group consisting of a pool of four different healthy equine subjects, each 3 years old.

### 3.2. SfMSCs Proliferation Assay

A stable arrest of the cell cycle is one of the hallmarks of aging [37]. In order to investigate the alterations in age-related cell proliferation, a BrdU assay was carried out. 

**Figure 3:**
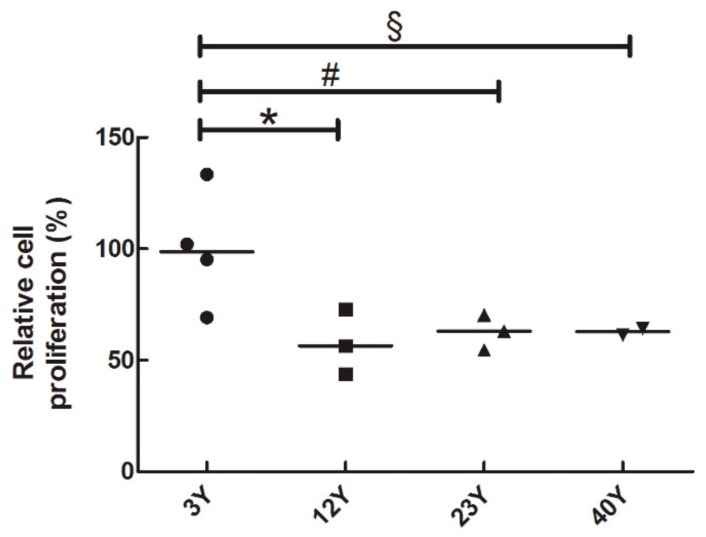
BrdU cell proliferation assay of equine SfMSCs cultured from donors at different ages. The values of each donor and the mean value (black line) inside each group are represented. All the data are expressed as percentages of relative amounts to a reference control group consisting of a pool of four different healthy equine subjects, each 3 years old. * represents a significant difference between 3Y and 12Y groups; *p* < 0.05. # represents a significant difference between 3Y and 22Y groups; *p* < 0.05. § represents a significant difference between 3Y and 40Y groups; *p* < 0.05.

Results showed a reduction in BrdU incorporation, during the S-phase of the cell cycle, in SfMSCs isolated in equine donors of 12Y, 23Y, and 40Y groups compared to young donors (3Y) (Figure 3). No significant age-related arrest of the BrdU incorporation was detected in any sample. 

### 3.3. Cell Cycle Analysis 

In order to better visualize any difference in the cell cycle, propidium iodide (PI) staining followed by flow cytometry analysis was carried out. 

**Figure 4:**
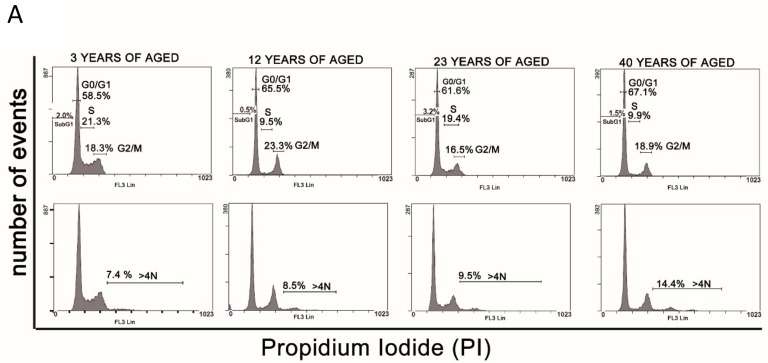

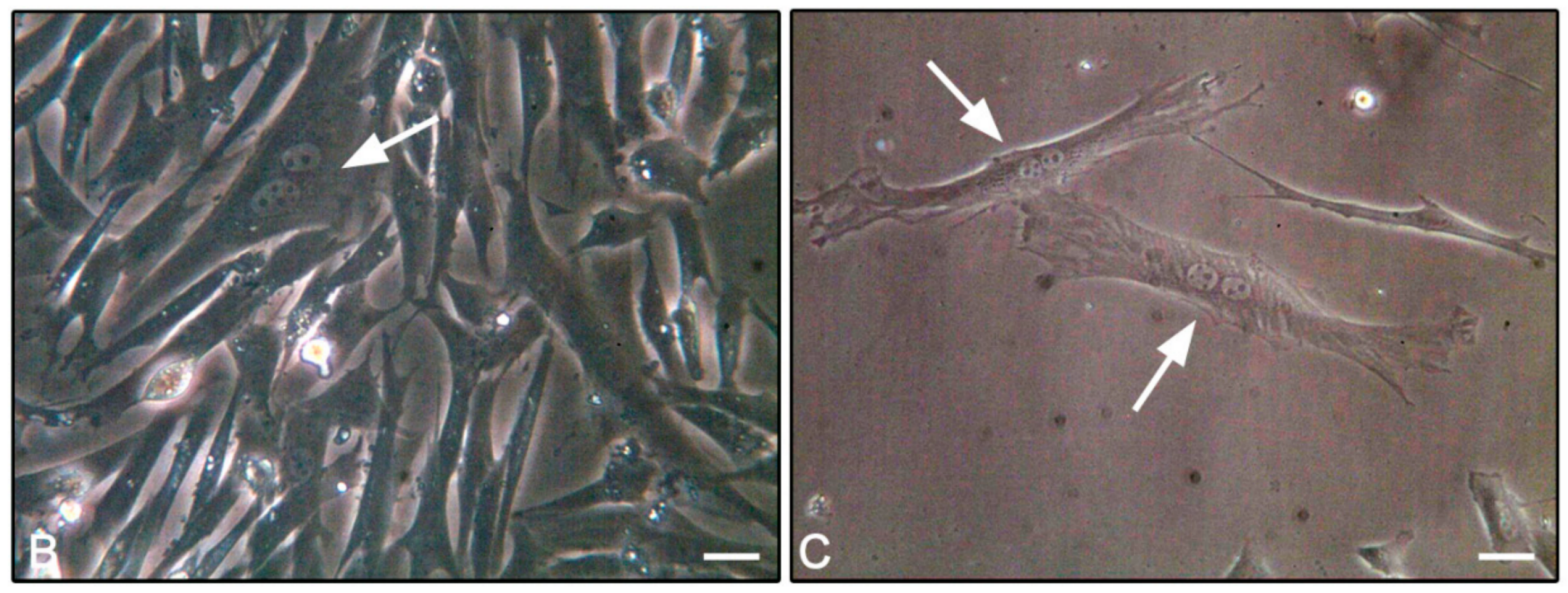
(**A**) Representative results of the cell cycle distribution of SfMSCs isolated from donors of different ages and measured by flow cytometry using propidium iodide staining. The percentage of the cell cycle the phase is shown in a bar graph form. A moderate gain of cells in the G0/G1 phase and a decrease of cells in S phase is observed when comparing the 3Y group with the 40Y group. An increasing number of age-related tetraploid cells is observed. Number of events: cell frequency; propidium iodide: DNA staining fluorochrome. (**B**) Representative light microscope images of in vitro SfMSCs isolated from a 3-year-old equine donor showing the presence of tetraploid cells (white arrow) (bar: 30 μm). (**C**) Representative light microscope image of in vitro SfMSCs isolated from a 40-year-old equine donor showing the presence of tetraploid cells (white arrows) (bar: 30 μm).

The flow cytometry analysis of PI-stained cells showed a moderate percentage increase of cells in the G0/G1 phase of the cell cycle, in a slightly age-dependent manner (Figure 4 and Table 3), in line with the reduction of proliferation rates already seen in the BrdU proliferation assay. Interestingly, these analyses also outlined an accumulation of tetraploid cells in all samples tested, and this feature was observed with an age-dependent increase in the percentage of tetraploid cells, ranging from 7.6% in young donors (3Y) to 14.4% in old donors (40Y) (Figure 4 A, Table 3 and Figure 4B). 

**Figure 5:**
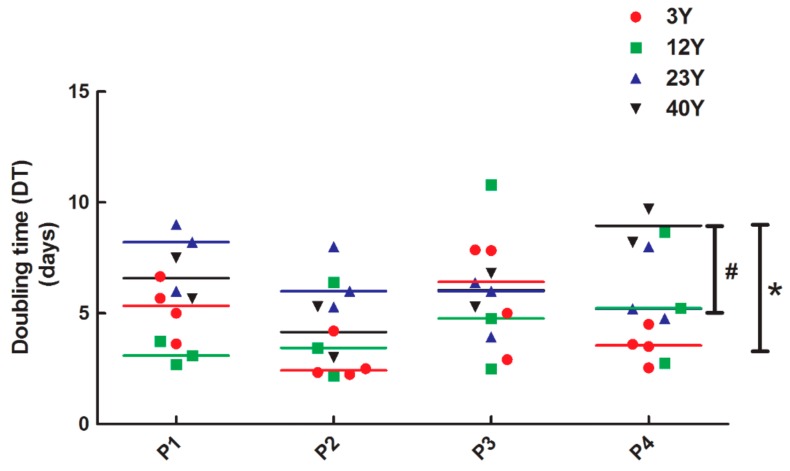
Doubling time for SfMSCs isolated from equine donors of different ages and cultured in vitro from passage 0 (P0) to passage 4 (P4). Values corresponding to each donor and the mean value (colored line) corresponding to each age-related group are reported. * represents a significant difference between 3Y and 40Y groups; *p* < 0.05. # represents a significant difference between 12Y and 40Y groups at P4; *p* < 0.05.

In order to better define the role of the cell cycle in senescence and aged cells, the doubling time assay was performed in SfMSCs derived from donors at different ages. The results showed a significant difference of the doubling time in SfMSCs cultured from 40Y donors (Figure 5) compared to 3Y and 12Y donors, in agreement with the previous results of the BrdU proliferation assay and flow cytometric analysis. However, the significant difference between the groups was observed only at passage 4 (p4). 

### 3.4. F-Actin Staining 

**Figure 6:**
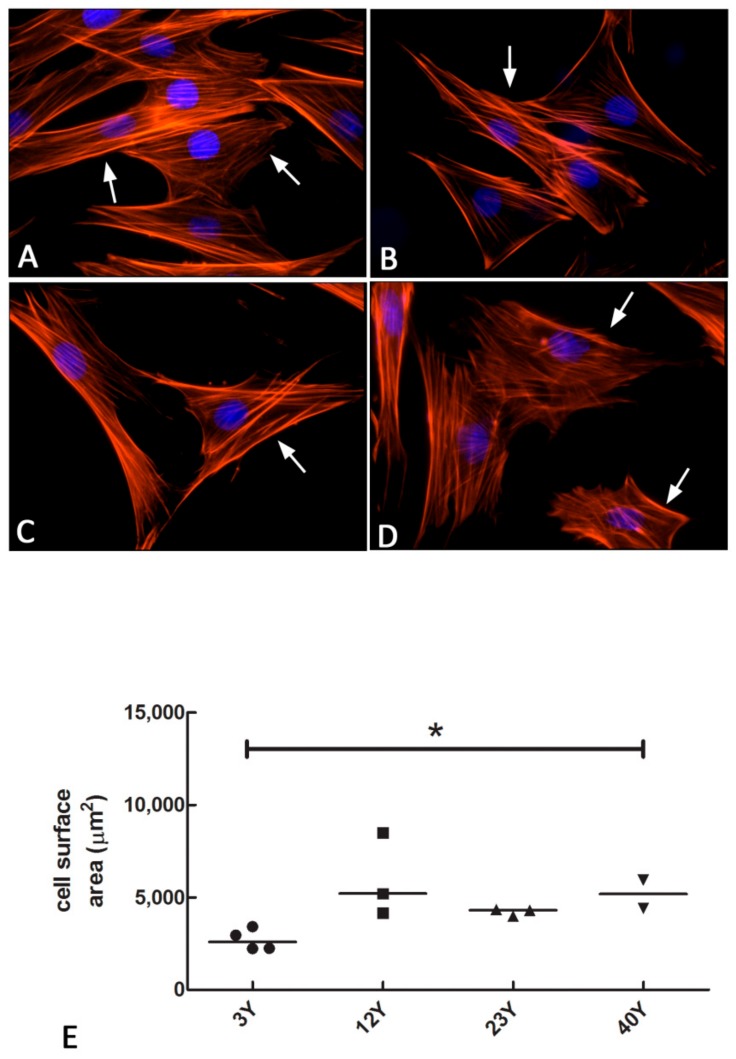
Fluorescence microscopy images of the phalloidin staining assay demonstrating the presence and distribution of actin fibers (red color; arrows) in equine SfMSCs isolated from donors of different ages. Nuclei were counterstained with DAPI (blue color). All the images have a magnification of 600× and are representative of three independent experiments. (**A**) SfMSCs isolated from equine donors of 3 years of age; (**B**) SfMSCs isolated from equine donors of 12 years of age; (**C**) SfMSCs isolated from equine donors of 23 years of age; (**D**) SfMSCs isolated from equine donors of 40 years of age. (**E**) Quantitative analysis of the cell surface area of equine SfMSCs isolated from donors at different ages and stained with phalloidin probe. Values corresponding to each donor and the mean value (line) corresponding to each age-related group are represented. * represents a significant difference between 3Y and 40Y groups; *p* < 0.05.

Since senescent cells stop dividing, they have significant alterations in their motility and strength, which reflect morphological changes due to a lack of integrity in the cytoskeleton. In order to demonstrate morphological changes connected with the expression and distribution of cytoskeleton proteins, the status of actin fibers in relation to the shape and size of cells was analyzed by phalloidin staining assay. Results showed the presence of several actin fibers, which are parallel-oriented in cells isolated from donors of 3-year-olds (3Y) (Figure 6A). A similar distribution and localization was observed in samples obtained from equine donors of 12 years of age (12Y) (Figure 6B), while a less regular orientation was detected in cells from equine donors of 23 years (23Y) (Figure 6C) and 40 years of age (40Y) (Figure 6D). In the latter sample, actin fibers showed a more random distribution, in combination with an enlarged cell size and irregular shape (Figure 6D). 

Since the cell surface area is one of the most pronounced morphological changes occurring in senescent cells, a quantitative analysis of the cellular area stained by the phalloidin probe was carried out. The results demonstrated an increase of cell surface area in SfMSCs isolated from the old donors (40Y) compared to young associated donors (3Y), confirming a relationship of the cell surface area with aging (Figure 6E). 

### 3.5. Ultrastructural Analysis of Equine SfMSCs 

To better evaluate the morphological changes associated with aging, an ultrastructural analysis by TEM was carried out. 

**Figure 7:**
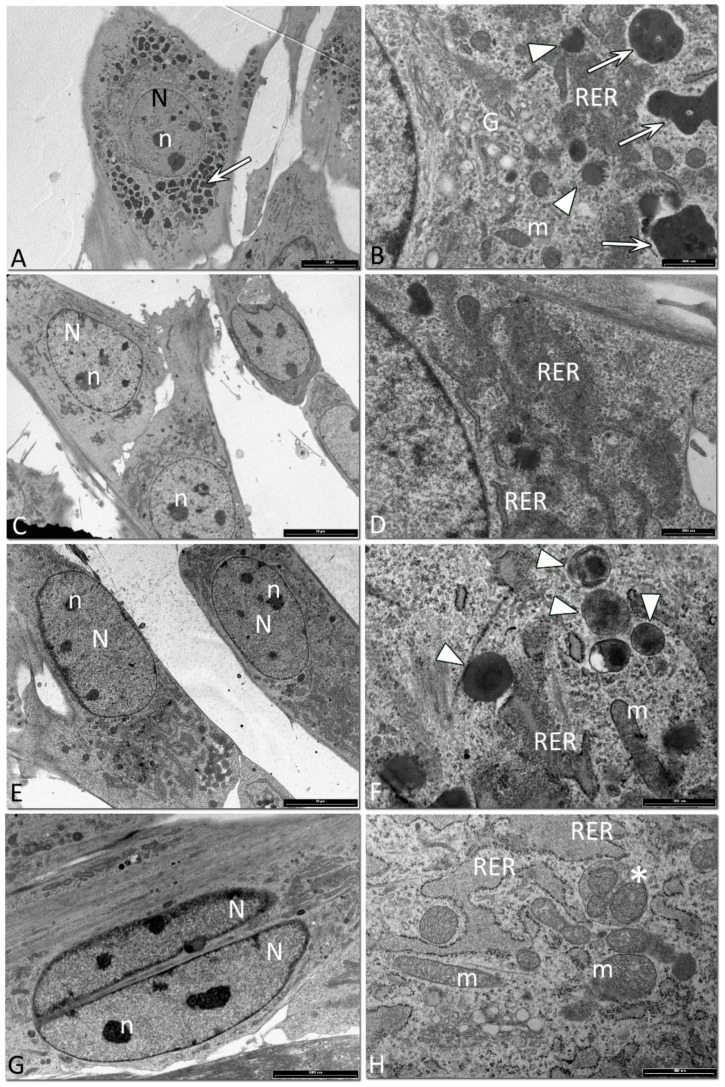
Representative images of the SfMSC ultrastructure. (**A**) Equine SfMSCs from a 3-year-old donor, showing a polygonal shape, with a round nucleus (N) and several nucleoli (n). The cytoplasm is characterized by the presence of several secondary lysosomes (white arrow) (bar: 10 μm). (**B**) Higher magnification of equine SfMSCs from a 3-year-old donor. A Golgi complex (G), mitochondria (m) and an enlarged rough endoplasmic reticulum (RER) are detected in the cytoplasm. Primary (arrowhead) and secondary lysosomes (white arrow) are observed (bar: 100 nm). (**C**) Equine SfMSCs from a 12-year-old donor. Cells showed a well-preserved nucleus (N) and nucleoli (n) and a widely developed RER, surrounding the nuclear envelope. There is a clear lack of secondary lysosomes (bar: 10 μm). (**D**) Cytoplasm details of equine SfMSCs from a 12-year-old donor, in which a regular RER and enlarged RER are clearly observed (bar: 1 μm). (**E**) Equine SfMSCs from a 23-year-old equine donor showing a fibroblast-like shape, regular nucleus (N), and several nucleoli (n) (bar: 10 μm). (**F**) Higher magnification of the cytoplasm of equine SfMSCs from a 23-year-old donor. Several primary lysosomes (arrowheads), mitochondria (m), and dilated RER are observed (bar: 1 μm). (**G**) Binucleated SfMSCs, corresponding to tetraploid cells, isolated from a 40-year-old equine donor showing a fibroblast-like shape, nucleus (N), and several nucleoli (n) (bar: 5000 nm). (**H**) Detail of the cytoplasm in which several mitochondria (m), a dilated RER, and mitophagy vesicles (*) are visible (bar: 1 μm).

Equine SfMSCs isolated from 3Y equine donors showed a round-shaped nucleus, with three or more well preserved nucleoli (Figure 7A). At low magnification, the cytoplasm appeared to be filled by several black vacuoles, corresponding to secondary lysosomes, suggesting a strong degradation activity (Figure 7A,B). At higher magnification, primary and secondary lysosomes were detected (Figure 7B). Regular mitochondria and a widely developed Golgi complex were observed (Figure 7B). An enlarged rough endoplasmic reticulum (RER) was detected, surrounding the nuclear envelope (Figure 7A,B). In equine SfMSCs, isolated from equine donors of 12 years of age, a reduced number of black vacuoles was observed (Figure 7C,D). At higher magnification, a few lysosomes and dilated RER cisternae were detected (Figure 7D). Equine SfMSCs, isolated from equine donors of 23-year-olds, showed a fibroblast-like shape, with a big nucleus and multiple nucleoli (Figure 7E). At higher magnification, several primary lysosomes, regular mitochondria, and dilated RER were observed (Figure 7F). Binucleated SfMSCs, corresponding to the tetraploid cells previously described, were often observed in donors of 40-year-olds (Figure 7G). At higher magnification, a dilated RER, several mitochondria, and several mitophagy vesicles were detected (Figure 7H). 

### 3.6. Chondrogenic Differentiation

**Figure 8:**
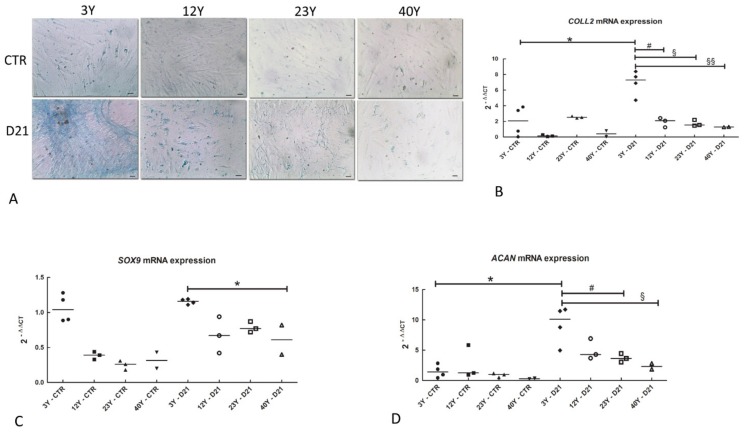
(**A**) Representative images of the Alcian blue staining assay showing the deposition of glycosaminoglycans (GAG) in the extracellular matrix in SfMSCs, isolated from equine donors of 3-year-olds (3Y), 12-year-olds (12Y), 23-year-olds (23Y), and 40-year-olds (40Y) and stimulated toward chondrogenic phenotypes for up to 21 days (scale bar: 30 μm). (**B**) Real-time PCR relative expression of *COLL2* mRNA in control (CTR) and differentiated (D21) SfMSCs toward chondrogenic phenotypes for up to 21 days. Values corresponding to each equine donor involved in the study and the mean value (black line) of each experimental group (3Y, 12Y, 23Y, and 40Y) are shown. Values are represented as fold increase related to a reference control group consisting of a pool of four different healthy equine subjects, each 3 years old. * represents a significant difference between 3Y CTR and 3Y D21 groups; *p* < 0.05. # represents a significant difference between 3Y D21 and 12Y D21 groups; p < 0.05. § represents a significant difference between 3Y D21 and 23Y D21 groups; *p* < 0.05. §§ represents a significant difference between 3Y D21 and 40Y D21 groups; *p* < 0.05. (**C**) Real-time PCR relative expression of *SOX9* mRNA in control (CTR) and differentiated (D21) SfMSCs toward chondrogenic phenotypes for up to 21 days. Values corresponding to each equine donor involved in the study and the mean value (black line) of each experimental group (3Y, 12Y, 23Y, and 40Y) are shown. Values are represented as fold increase related to a reference control group consisting of a pool of four different healthy equine subjects, each 3 years old. * represents a significant difference between 3Y D21 and 40Y D21 groups; *p* < 0.05. (**D**) Real-time PCR relative expression of *ACAN* mRNA in control (CTR) and differentiated (d21) SfMSCs toward chondrogenic phenotypes fir up to 21 days. Values corresponding to each equine donor involved in the study and the mean value (black line) of each experimental group (3Y, 12Y, 22Y, and 40Y) are shown. Values are represented as fold increase related to a reference control group consisting of a pool of four different healthy equine subjects, each 3 years old. *P* < 0.05. * represents a significant difference between 3Y CTR and 3Y D21 groups; *p* < 0.05. # represents a significant difference between 3Y D21 and 23Y D21 groups; *p* < 0.05. § represents a significant difference between 3Y D21 and 40Y D21 groups; *p* < 0.05.

In order to demonstrate the deposition of glycosaminoglycans (GAG) in the extracellular matrix of SfMSCs, isolated from equine donors at different ages, the Alcian blue staining procedure was carried out. Figure 8A shows the presence of an intense blue color in the extracellular matrix of SfMSCs isolated from equine donors of 3 years of age and stimulated in vitro toward chondrogenic phenotypes for up to 21 days (Figure 8A, D21 3Y). A gradual decrease of the color intensity was observed in SfMSCs isolated from 12 and 22-year-old equine donors (Figure 8A). Samples obtained from equine donors of 40 years of age did not show any kind of color, suggesting a low amount of GAG deposition (Figure 8A). 

To demonstrate a modified age-related ability of SfMSCs to differentiate toward chondrogenic phenotypes, the expression of mRNA chondrogenic markers was analyzed after 21 days of in vitro stimulation with chondrogenic factors. 

In control samples of SfMSCs isolated from all donors, a low level of *COLL2A* mRNA was detected (Figure 8B), while the level of *COLL2A* mRNA was consistently higher in 3-year-old equine SfMSCs stimulated to chondrogenic differentiation for up to 21 days (Figure 8B). Furthermore, in chondrogenic differentiated samples, *COLL2A* mRNA showed a strong age-related reduction (Figure 8B).

Regarding the transcription factor *SOX9*, real-time PCR data demonstrated a weak upregulation in all the differentiated samples (Figure 8C) compared to their corresponding control groups, in agreement with the long chondrogenic stimulation (21 days). However, the highest level of expression was shown in the 3Y D21 group, followed by a decrease of the signal in all the other differentiated groups (Figure 8C), suggesting age-related *SOX9* mRNA expression. 

SfMSCs showed a weak signal of *ACAN* mRNA in all the control groups (Figure 8D), while a strong upregulation was detected in all the chondrogenic differentiated groups (Figure 8D). Similar to *SOX9* mRNA expression, the highest signal of *ACAN* mRNA expression was observed in the 3Y D21 group, followed by a gradual reduction in all the other differentiated groups, suggesting an age-dependent relationship of *ACAN* mRNA expression. 

## 4. Discussion

Osteoarthritis is a degenerative disease that strongly correlates with age [38,39]. Mesenchymal stem/progenitor cells derived from synovial joint tissues exhibit a superior chondrogenic ability compared with those derived from non-joint tissues [1,40,41]; thus, they represent the best choice for the regeneration and repair of cartilage [40]. Nevertheless, aging can compromise the biological proprieties of MSCs and their ability to differentiate into multiple cell types of the mesoderm germ layer [29]. 

In this study, we focused our attention on equine SfMSCs and some age-related alterations, which influence their tissue repair abilities. The main goal was to demonstrate the presence of an increasing amount of age-related hallmarks in synovial fluid MSCs and their influence on the potential ability to differentiate toward chondrogenic phenotypes. 

Sfs from healthy equine donors of different ages were collected and MSCs were isolated according to previous protocol [27]. We decided to base our study on an equine model due to the non-invasive technique of synovial fluid collection and because horses represent a useful animal model of several spontaneous disorders that are clinically relevant to similar human disorders [19]. Furthermore, joint injuries are among the most common catastrophic injuries in thoroughbred racehorses, which are mainly repaired by arthroscopic surgery [42].

All the SfMSCs tested in our study were positive for the mesenchymal cell surface markers *CD90*, *CD105*, while they were negative for *CD34* [19,35,43]. Furthermore, real-time PCR data demonstrated the lack of significant quantitative differences in the expression of the *CD90* and *CD105* between young and old donors, suggesting that there is no relationship between mesenchymal surface marker expression and age. A similar trend was observed for the expression of multipotency markers. Sf-MSCs isolated from equine donors of different ages were positive for *OCT4, NANOG*, and *SOX2* mRNA expression, suggesting that even healthy, age-derived MSCs keep their mesenchymal and self-renewal phenotype. The lack of a quantitative difference in *OCT4* mRNA expression confirms these results, while an upregulation of *NANOG* and *SOX2* mRNA expression was unexpectedly observed in equine age-derived SfMSCs (40Y group). A few data are available regarding the variation of the expression of cluster of differentiation markers and self-renewal markers related to aging in stem cells [44,45]. Human MSCs isolated from adipose tissue express pluripotency markers over several in vitro passages [37]. In addition, it has been demonstrated that a high level of *NANOG* and *OCT4* protein expression in adult MSCs is essential for maintaining the self-renewal properties of MSCs [46]. All in all, our data demonstrate the lack of a relationship between the expression of mesenchymal stem cells and multipotency markers with aging, and the ability to isolate adult stem cells even from healthy aged donors. Although previous studies on age-related alterations of stem cells demonstrated stem cell exhaustion as a hallmark of aging [44], this is not present in hematopoietic stem cells, which show an increase in stem cell proliferation related to aging [38]. 

The BrdU cell proliferation assay showed a 50% reduction of cell proliferation in SfMSCs isolated from 40-year-old horses compared to 3Y donors, suggesting the acquisition of a gradual senescent phenotype, one of the most repetitive hallmarks of aging [38,44]. These results are confirmed by a doubling time assay, which showed the highest time for cell doubling in the 40Y sample. 

The flow cytometry analysis of equine SF-MSCs, surprisingly, demonstrated the presence of tetraploid cells in all samples analyzed. However, the percentage increased with age, suggesting a relationship between tetraploidy and aging. Although nearly all mammalian species are diploid, whole genome duplications occur in select mammalian tissues as part of normal development [47]. In humans, a programmed polyploidization has been described in the placenta [48], which can reach a DNA content of up to 64 N. Megakaryocytes [49] and hepatocytes [50] become polyploid through abortive mitosis, while in skeletal muscles and osteoclasts, cell fusion generates polynucleated terminally differentiated cells [51]. To date, the benefit of programmed polyploidy is not fully understood. However, the presence of tetraploid and/or octaploid cells in some tissues correlated with age or stress conditions has been described [47,48]. The incidence of polyploidy increases with age in hepatocytes and vascular smooth muscle cells [50,52], while cardiomyocytes under cardiac overloading and hypertension [53] and hepatocytes under oxidative stress [54] showed a high level of polyploid cells. The mechanisms and consequences of polyploidy in pathological situations are still unknown [47]. Estrada and colleagues have demonstrated the presence of a high percentage of aneuploid MSCs, which progressively increase until senescence [55], proposing aneuploidy as a main cause of genetic instability and replicative senescence and suggesting the assessment of aneuploidy before any clinical use of MSCs.

Since senescent cells cease to divide and have marked changes in morphology, motility, and mechanical strength, the integrity of cytoskeletons is expected to be modified [56]. Since actin stress fiber is a key factor determining cell shape and motility, a change in its abundance and localization may be causatively related to the enlarged and round shape as well as the decreased motility of senescent cells [56]. By using phalloidin staining assay, a selective probe for F-actin, the presence and localization of actin filaments has been investigated in aged equine SfMSCs. The results showed the presence of parallel filaments in young SfMSCs, while a more irregular and intermingled disposition of actin fibers was observed in aged SfMSCs. The resulting cellular shape was largely modified in age-derived cells compared to young cells, changing from short spindle fusiform cells to a large flat spread. Moreover, the cellular surface area greatly increased in age-derived MSCs, in agreement with previous studies carried out on dermal age-derived MSCs, where the increase in the surface area was attributed in part to changes in the cytoskeleton [57]. 

Alterations of subcellular organelles such as lysosomes, mitochondria, nuclei, and the endoplasmic reticulum (ER) has been already described in senescent cells [56]. The ultrastructural analysis of equine SfMSCs showed the presence of several autophagic vesicles, a widely dispersed endoplasmic reticulum and Golgi complex, several lysosomes, and an autophagosome—all morphological features connected with active stem cells, which require these functions to get rid of cellular waste produced during their quiescent stage [58]. Autophagy is a highly conserved catabolic process whereby intracellular components, such as protein, organelles, and cytoplasm, are delivered to lysosomes for self-degradation. The final goal is to maintain cellular homeostasis under physiological and stress conditions. Its activation promotes cellular survival by maintaining adequate metabolic functions, bioenergetics levels, and an amino acid pool. Being a sensor of stress, autophagy has been linked to aging [58]. Several studies on animal models [59] and human tissues, such as muscle and hematopoietic stem cells, support a decline in autophagic activity related to aging [58,60]. Our analysis demonstrated a huge number of primary lysosomes and autophagosomes in the cytoplasm of youth-derived cells, while they disappeared in cells isolated from donors of 40 years of age, confirming a decline of autophagy activity during aging. Furthermore, ultrastructure observations demonstrated a widely distributed RER inside the cytoplasm in youth-derived cells, suggesting an intense RER activity connected with the maintenance of cellular homeostasis. On the contrary, age-derived cells showed a dilated RER morphology, surrounded by a reduced number of ribosomes, suggesting the presence of ER stress, as a consequence of an imbalance between ER protein folding loads and capacity, leading to the accumulation of unfolded proteins in the ER lumen [61,62]. One counteraction to the cellular microenvironment induced by ER stress is the activation of mitophagy, a particular kind of autophagy, the aim of which is to destroy mitochondria and, in such a way, to protect against the release of pro-apoptotic proteins, the generation of toxic reactive oxygen species (ROS), and the futile hydrolysis of adenosine triphosphate (ATP) [63]. In our samples, ER stress, coupled to several mitophagosomes, was distinctly observed only in SfMSCs isolated from equine donors of 40 years of age, confirming an activation of mitochondrial autophagy in aged MSCs. 

Aging can seriously compromise the ability of MSCs to differentiate in vitro towards osteogenic, adipogenic, and chondrogenic phenotypes. With SfMSCs being more prone to chondrogenic differentiation [40], we investigated the correlation between age-related SfMSCs and chondrogenic ability. To this end, SfMSCs were stimulated in vitro toward chondrogenic differentiation for up to 21 days. Alcian blue staining assay and real-time PCR for chondrogenic markers showed a reduced ability of SfMSCs, isolated from equine donors of 40 years of age, to differentiate toward chondrogenic phenotypes, suggesting an impairment of chondrogenic differentiation in aged SfMSCs [64]. 

## 5. Conclusion

Differences in the characteristics of stem cells derived from donors of different ages can affect the outcome of cell-based therapy [64]. 

In this study, SfMSCs did not show any significant alteration in their mesenchymal phenotype and multipotency related to aging, suggesting an unmodified ability in their biological proprieties and differentiation ability. Nevertheless, some senescence-related features such as cell growth, cell surface area, and cell ultrastructure demonstrate gradual alterations connected with aging, which could take part in the impairment of the functionality of MSCs. Indeed, our data demonstrate that the main feature compromised by aging is the ability of SfMSCs to differentiate toward chondrogenic phenotypes, which results in significantly downregulated chondrogenic markers in age-derived SfMSCs. 

These results confirm that aging can significantly compromise MSCs’ characteristics and performance, which should be taken into consideration in regenerative medicine based on MSC application. 

## Figures and Tables

**Table 1:** Profiles of Sf samples obtained from the joints of horses of different ages.

	Breed	Age (Years)	Gender	Donor’s Number
1	Standardbred	3	M	4
2	Standardbred	12	F	2
3	Pleasure riding horse	12	M	1
4	Standardbred	23	F	3
5	Pleasure riding horse	40	M	2

**Table 2:** Real-Time PCR primer sequences.

Name	Forward	Reverse	bp
*CD90*	5′-ATGAGAATACCACCGCCACA-3′	5′-AGTTTGTCTCGGAGCACAGA-3′	262
*CD105*	5′-TCAGGTCCCCAACACTAACC-3′	5′-AGTCTTGTTCGTGCTGAGGA-3′	148
*CD34*	5′-CCTTGCCCAGTCTGAGGTTA-3′	5′-GTCTTGCGGGAATAGTGCTG-3′	172
*SOX9*	5′-GAACAGCCCGTCTACACACA-3′	5′-GCCACTGATTCGCAACAAGG-3′	235
*COLL2*	5′-CTGGCAAGCAAGGAGACAGA-3′	5′-CCATTAGCGCCATCTTTGCC-3′	292
*ACAN*	5′-TCATCTAGAGCCCACTGCCT-3′	5′-AGTCCACCGAGGTCCTCTAC-3′	234
*NANOG*	5′-TCTCTCCTCTGCCTTCCTCC -3′	5′-TCTGCTGGAGGCTGAGGTAT-3′	225
*OCT4*	5′-GGTACGAGTGTGGTTCTGCA-3′	5′-ACCGAGGAGTACAGCGTAGT-3′	192
*SOX2*	5′-GCCCTGCAGTACAACTCCAT-3′	5′-GACTTGACCACCGAACCCAT-3′	128
*GAPDH*	5′-TGCCCCAATGTTTGTGATGG-3′	5′-CACTGTGGTCATGAGTCCCT-3′	154

**Table 3:** Flow cytometry data of cell cycle distribution and frequency of tetraploid cells (4N) in SfMSCs isolated from equine donors of different ages.Values corresponding to each equine donor are reported and organized in the experimental group associated with age. The experiment was repeated in triplicate for each donor, and the results are expressed as percentage ± SD. ^a^ represents a significant difference from 40Y group; *p* < 0.05.

	G0/G1 (%)	S Phase (%)	G2/M (%)	4N (%)
**3Y Group**				
Donor 1	56.6 ± 1.2	5.3 ± 2.6	28.6 ± 1.4	8.6 ± 1.2 ^a^
Donor 2	58.0 ± 1.1	9.7 ± 1.4	26.9 ± 1.6	7.5 ± 0.9 ^a^
Donor 3	58.5 ± 1.4	21.3 ± 0.9	18.3 ± 1.1	7.4 ± 0.9 ^a^
Donor 4	48.6 ± 0.8	15.0 ± 0.5	28.2 ± 0.9	7.6 ± 0.3 ^a^
**12Y Group**				
Donor 1	64.1 ± 1.6	10.1 ± 1.1	23.3 ± 1.4	12.0 ± 1.2
Donor 2	65.6 ± 1.6	9.5 ± 1.5	23.3 ± 1.1	8.5 ± 0.9
Donor 3	59.9 ± 1.7	9.0 ± 1.6	22.6 ± 1.2	8.2 ± 0.5
**23Y Group**				
Donor 1	68.7 ± 1.6	4.9 ± 0.9	25.5 ± 1.4	10.5 ± 0.6
Donor 2	61.6 ± 0.5	9.4 ± 0.2	16.5 ± 0.6	9.5 ± 0.3
Donor 3	84.7 ± 0.8	7.8 ± 0.3	5.2 ± 0.7	10.7 ± 0.3
**40Y Group**				
Donor 1	67.1 ± 1.1	9.9 ± 1.5	18.9 ± 1.2	14.4 ± 0.7
Donor 2	74.3 ± 1.2	8.1 ± 2.4	16.1 ± 1.5	18.5 ± 1.1
